# ﻿The terrestrial isopod fauna (Isopoda, Oniscidea) of Abrau Peninsula, north-west Caucasus, Russia

**DOI:** 10.3897/zookeys.1225.121048

**Published:** 2025-02-05

**Authors:** Daria M. Kuznetsova, Konstantin B. Gongalsky

**Affiliations:** 1 Severtsov Institute of Ecology and Evolution of the Russian Academy of Sciences, Moscow 119071, Russia Severtsov Institute of Ecology and Evolution of the Russian Academy of Sciences Moscow Russia; 2 Present address: Edinburgh, UK Unaffiliated Edinburgh United Kingdom

**Keywords:** Checklist, database, inventory, soil fauna, soil macrofauna

## Abstract

From 2001 to 2022, the woodlice fauna of Abrau Peninsula, north-west Caucasus, Russia was examined. The collections yielded 5,581 specimens, which belong to 25 species, 19 genera, and 15 families. The most diverse families are Cylisticidae, Platyarthridae, Trachelipodidae, and Trichoniscidae, each with three species. The most diverse genera are *Platyarthrus* and *Trachelipus*. Five species have been noticed to the Russian fauna for the first time: *Tyloseuropaeus*, *Acaeroplastesmelanurus*, *Platyarthruscaudatus*, *Buddelundiellacataractae*, and Armadillidiumcf.marmoratum. The fauna of the Abrau Peninsula is predominantly Mediterranean and with a high percentage of endemics (12%). Further records are expected with more detailed studies of especially the family Trichoniscidae.

## ﻿Introduction

Mediterranean ecosystems are among the most disturbed in the world (Mouilliot et al. 2002), but they are also one of the possible centers of origin of woodlice, which have a high diversity here (Taiti et al.1998; Sfenthourakis and Taiti 2015). Due to this, the Mediterranean ecosystems need both research and conservation. In the territory of Russia, sub-Mediterranean ecosystems are widespread in the north-west Caucasus. Abrau Peninsula is a unique well-preserved area of sub-Mediterranean ecosystems on the Black Sea coast, and the Navagir Ridge separates it from the main transportation lines, making it almost inaccessible to tourists. It serves as a reference for comparable regions around the Black Sea coast. The peninsula is currently protected by a nature reserve, making understanding of its wildlife particularly important.

The soil fauna have been intensively explored in Russia’s Mediterranean ecosystems. K.V. Arnoldi and M.S. Gilyarov began exploring forest ecosystems between Novorossiysk and Dzhankhot beginning in the 1950s (Arnoldi and Gilyarov 1958; Gilyarov 1965, 1972). Later, owing to the efforts of A.D. Pokarzhevskii, thorough research was conducted in the Utrish State Nature Reserve, which was established on the Abrau Peninsula in 2010 (Gongalsky et al. 2004, 2006; Korobushkin 2014).

Woodlice (Isopoda, Oniscidea) are among the most common soil biota in these ecosystems. Our initial attempts to identify the material were rather unsuccessful due to the lack of proper keys, but with the assistance of major specialists in woodlice taxonomy (see Acknowledgements), we were able to produce an up-to-date identification of species. Based on this, both faunal (Gongalsky 2017, 2022; Gongalsky et al. 2024) and ecological publications (Gongalsky and Kuznetsova 2011) on woodlice in the region have been published. The advancement of molecular genetic technologies has lately allowed for the clarification of the status of some dubious species, and now this work has some horizons that bring us closer to understanding the woodlice fauna of the Abrau Peninsula. However, there are still plenty of opportunities for work in this region, and the proposed list is far from complete. Our goal is to compile all woodlice findings made on Abrau Peninsula to this time and conduct a preliminary analysis of its fauna.

## ﻿Materials and methods

### ﻿Study area

**Figure 1.**
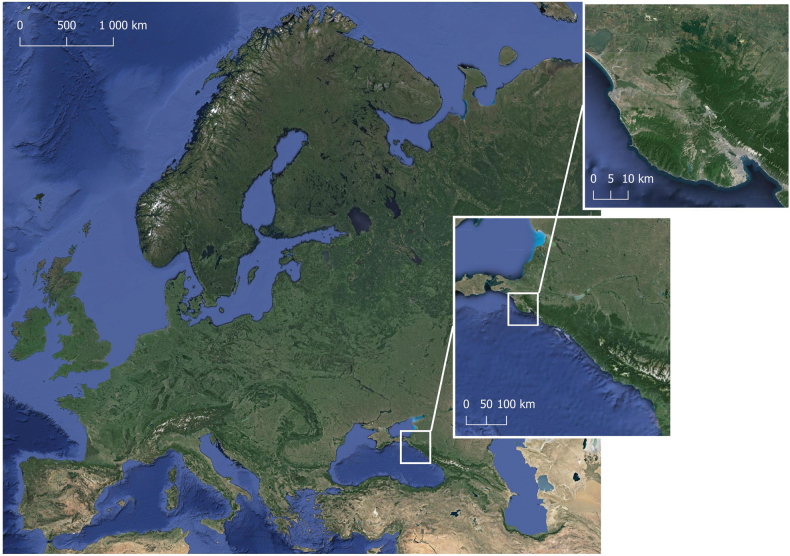
Map of the location of the study area (Abrau Peninsula).

**Figure 2.**
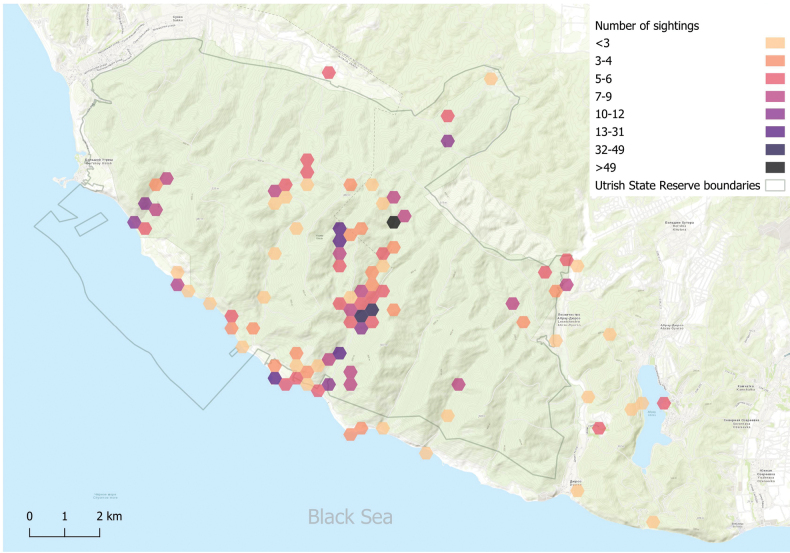
Map of observations.

Studies of the woodlice fauna on Abrau Peninsula were conducted between 2001 and 2022. For sampling, we used the most diverse biotopes and the maximal diversity of microhabitats within each of them (Figs 1, 2).

### ﻿Sampling methods

The resulting database of isopods location records comprised both individual faunal discoveries and finds from soil macrofauna surveys. All species were collected using hand-sorting from the litter and upper soil layer (down to a depth of 10–15 cm).

All collected animals were fixed in alcohol for subsequent laboratory identification. The biotope parameters, location, and date of sample were documented. Isopod species were identified using original descriptions (Borutzky 1948, 1977) and monographs on terrestrial isopods in Europe (Vandel 1960, 1962; Gruner 1966; Schmolzer 1965; Schmidt 1997; Sutton 2013). The taxonomy follows Schmalfuss (2003).

### ﻿Description of the database

A database of Abrau Peninsula isopod faunistic sightings was published in GBIF (https://doi.org/10.15468/zdypk6). It has 639 occurrences of 5,581 specimens from 25 species. The dataset covers the period from 2001 to 2022. Each entry includes the species, the names of the collector(s) and identifiers, geographical coordinates, the date (at least the month and year), and the number of specimens collected.

### ﻿Analysis of the fauna

We used the range classification proposed by Schmalfuss (2003). Since many range types are more detailed, they are grouped under these five categories: (i) Abrau endemics, (ii) Caucasus (Caucasus; North Caucasus), (iii) Mediterranean (Black Sea coast; Mediterranean and Black Sea coasts; Mediterranean, Black Sea costs and Middle Asia; Mediterranean, Atlantic and Black Sea coasts; Mediterranean and Atlantic coasts; European Mediterranean and Atlantic coasts; Macaronesian, Mediterranean and Black Sea coasts; Mediterranean), (iv) Europe (Europe, North Africa, Asia Minor; Europe, Asia Minor; Europe), (v) Cosmopolitan (Coastal cosmopolitan).

## ﻿Results

The database contains 639 records for 5,581 individuals of woodlice. The woodlice fauna of the Abrau Peninsula includes 25 species from 19 genera and 15 families.

### ﻿A commented list of terrestrial isopods of the Abrau Peninsula

First record for the Russian fauna is indicated by an asterisk (*).

Family Ligiidae Leach, 1814

1. *Ligidiumfragile* Budde-Lund, 1885 – Western Caucasus. Hygrophilic. Widely distributed in the western Caucasus. Occurs along the banks of streams and freshwater reservoirs, not more than a few meters from the water. Number of records: 50.

Family Tylidae Dana, 1852

* *Tyloseuropaeus* Arcangeli, 1938 – East Atlantic and Mediterranean. Coastal halophilic. The species has a wide eastern Mediterranean distribution, but was overlooked in the material from the Abrau Peninsula. It prefers stony seashores. Number of records: 2.

Family Buddelundiellidae Verhoeff, 1930

* *Buddelundiellacataractae* Verhoeff, 1930 – European. Mesophytic forests. The species inhabits deep moist litter, mainly in broadleaf forests. There is high diversity within this genus (Gardini and Taiti 2023), so molecular analysis is welcomed to check the presence of cryptic species on the Abrau Peninsula. Number of records: 6.

Family Trichoniscidae G.O. Sars, 1899

1. *Caucasocyphoniscustaitii* Gongalsky, 2022 – Abrau Peninsula (endemic). Hygrophilic. This species was once recorded in 2004 as single individual in thick leaf litter of broadleaf forests and then found again in high numbers in 2017–2020 in water oozing from cracks of cliffs. This presumes that this is an inhabitant of MSS (milieu souterrain superficiel). Number of records: 3.
2. *Haplophthalmusdanicus* Budde-Lund, 1880 – European, now cosmopolitan. Hygrophilic, this species inhabits deep moist litter, mainly in broadleaf forests. Number of records: 28.
3. *Trichoniscuspygmaeus* Sars, 1898 – Atlantic–Mediterranean. Hygrophilic. The species inhabits the banks of streams and freshwater reservoirs and no more than a few metres from the water’s edge. Given the high diversity within the genus in Europe, as well as the presence of other species of the genus on the Black Sea coast of the Caucasus, molecular genetic analyses are needed to identify potential cryptic species in this genus on the Abrau Peninsula. Number of records: 68.

Family Philosciidae Kinahan, 1857

1. *Chaetophilosciahastata* Verhoeff, 1928 – Eastern Mediterranean–Central Asian. Xerophilic. One of the main isopod species of Mediterranean shrublands (consisting of several species of juniper, pistachio, and Jerusalem thorn). This species was included in the regional Red Data Book of Krasnodar Krai as an indicator species of Mediterranean shrublands forests. Number of records: 40.

Family Halophilosciidae Verhoeff, 1908

1. *Halophilosciacouchii* (Kinahan, 1858) – Mediterranean–Atlantic–Black Sea coasts by origin, but now almost cosmopolitan. Coastal halophilic. A terrestrial isopod that lives on the sea coast within a few meters of the water’s edge. Number of records: 7.

Family Platyarthridae Verhoeff, 1949

* *Platyarthruscaudatus* Aubert & Dollfus, 1890 – Mediterranean. Myrmecophilic. Detected only in recent faunal studies due to the mass survey of anthills. The species corresponds to the type description. Number of records: 3.
2. *Platyarthrushoffmannseggii* Brandt, 1833 – Europe–North Africa–Asia Minor by origin, but now Holarctic. Myrmecophilic. The species is widespread throughout the peninsula, both in anthills located in Mediterranean vegetation and a few kilometres from the sea in the belt of broadleaf forests. Number of records: 11.
3. *Platyarthrusschoblii* Budde-Lund, 1885 – Macaronesian–Mediterranean–Black Sea coasts. Myrmecophilic. Despite the wide distribution of the species along the Black Sea coast, it was found in only one locality, in an anthill near a settlement. Probably spread by humans. Number of records: 1.

Family Agnaridae Schmidt, 2003

1. *Protracheoniscuskrivolutskyi* Gongalsky, 2024 – Abrau Peninsula (endemic). Eurytopic. A common and numerous species on the peninsula. It dominates in many ecosystems, in particular, *Quercuspetraea* oak forests. This species inhabits lowlands, slopes, and tops of gorges, and it may be the only species in upland communities. Until recently it was attributed to *Protracheoniscusfossuliger* (Verhoeff, 1901), but closer examination of morphology and application of molecular genetic markers revealed the authenticity of this species (Gongalsky et al. 2024). Number of records: 105.

Family Cylisticidae Verhoeff, 1949

1. *Cylisticusconvexus* (De Geer, 1778) – Almost cosmopolitan. Mesophitic forests. This species is mainly distributed in the settlements of Abrau and Maly Utrish, but it also occurs in forests. Molecular genetic data confirm that our material belongs to this species (data not published). Number of records: 13.
2. *Cylisticusgiljarovi* Borutzky, 1977 – Northern Caucasus. Mesophitic forests. This species occurs exclusively beyond the Navagir Ridge, on the northern macro-slope of the ridge separating these ecosystems from the sea. It inhabits oak and hornbeam forests. The present finding is the second mentionioning of the species since its description (Borutzky 1977). Number of records: 2.
3. *Parcylisticusdentifrons* (Budde-Lund, 1885) – Northern Caucasus and northern Pre-Caspian. The species is probably at the edge of its range; it inhabits mainly the Caucasus. In addition to the Abrau Peninsula, it also occurs in Novorossiysk. Number of records: 1.

Family Porcellionidae Brandt, 1831

* *Acaeroplastesmelanurus* (Budde-Lund, 1885) – Mediterranean and East Atlantic distribution. Mesophitic forests. Found exclusively behind Navagir Ridge, on the northern macro-slope of the ridge separating these ecosystems from the sea. Occurs in oak and oriental hornbeam forests. Fully corresponds to the type description. Number of records: 2.
2. *Porcellionidespruinosus* (Brandt, 1833) – Mediterranean, but now cosmopolitan. Synanthropic. The species prefers forest-steppe and steppe territories. Where detected on the Abrau Peninsula, the sites have mainly synanthropic habitats. Number of records: 5.

Family Trachelipodidae Strouhal, 1953

1. *Trachelipuslutshniki* (Verhoeff, 1933) – Western-Caucasus. Mesophitic forests. The species has been described from the environs of Sochi, and the present find is the westernmost point, considerably extending its range. Fully corresponds to the type description. Number of records: 2.
2. *Trachelipusrazzautii* Arcangeli, 1913 – Mediterranean. Mesophitic forests. One of the most widespread species of isopods on the Abrau Peninsula and is found almost everywhere. Probably, may be a cryptic species; therefore, a comparison using molecular genetic markers with material from the topotype of *T.razzautii* is necessary. Number of records: 102.
3. *Trachelipusutrishensis* Gongalsky, 2017 – Abrau Peninsula (endemic). Mesophitic forests. It is a recently described species, which inhabits only broadleaf forests on the peninsula. Number of records: 41.

Family Detonidae Budde-Lund, 1904

1. *Armadilloniscusellipticus* (Harger, 1878) – Almost cosmopolitan. Coastal halophilic. Occurs in the littoral areas. Prefers rocks in the tidal zone. Number of records: 15.

Family Armadillidiidae Brandt, 1833

* *Armadillidium* cf. *marmoratum* Strouhal, 1929 – Mediterranean. Coastal halophilic. This species was found only on beaches. It is probably a new species, as identification in collaboration with S. Taiti resulted in an unclear diagnosis. Molecular genetic analysis and thorough morphological study are required, which is our planned. Number of records: 7.
2. *Armadillidiumvulgare* (Latreille, 1804) – Mediterranean, but now cosmopolitan. Euryoecious. This is one of the most common and largest species on the peninsula. Number of records: 103.

Family Armadillidae Brandt, 1831

1. *Armadilloofficinalis* Duméril, 1816 – Mediterranean. Xerophilic. One of the main inhabitants of Mediterranean shrublands (consisting of several species of juniper, pistachio, and Jerusalem thorn). This species was Included in the regional Red Data Book of Krasnodar Krai as an indicator species of Mediterranean shrublands. It has been found in the settlement of Malyi Utrish. Number of records: 20.

Incertae sedis

1. *Buchnerillolittoralis* (Verhoeff, 1942) – Mediterranean. Hygrophilic. The species was captured between stones in a vertical wall at the Zhemchuzhnyi waterfall, the place where the watercourse pours out of the crevasse into the sea. The waterfall is located tens of meters from the tide line. Due to the displacement of the waterfall in the last few years, findings have ceased. Perhaps the species was introduced here, survived for a few years, and then disappeared. Number of records: 2.

In Abrau Peninsula, there are three endemic species: *Caucasocyphoniscustaitii*, *Trachelipusutrishensis*, and *Protracheoniscuskrivolutskyi*.Four other species were defined as the Caucasus endemics: northern Caucasus *Cylisticusgiljarovi* and *Parcylisticusdentifrons*, and western Caucasus *Ligidiumfragile* and *Trachelipuslutshniki*. For last species, the Abrau Peninsula record is the westernmost and, thus, extend this species’ range substantially.

**Figure 3.**
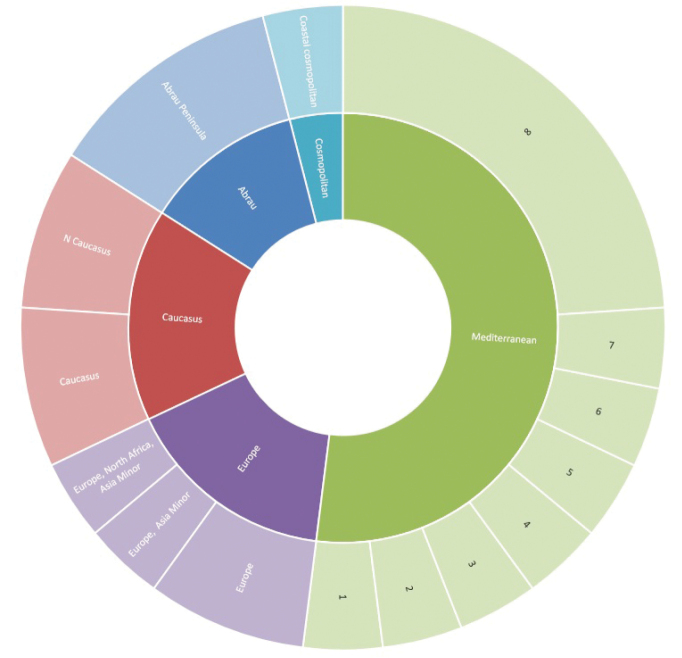
Types of woodlice species original distribution of Abrau Peninsula based on number of species. Captions: 1 – Black Sea coast; 2 – Mediterranean and Black Sea coasts; 3 – Mediterranean, Black Sea costs, and Central Asia; 4 – Mediterranean, Atlantic, and Black Sea coasts; 5 – Mediterranean and Atlantic coasts; 6 – European Mediterranean and Atlantic coasts; 7 – Macaronesian, Mediterranean, and Black Sea coasts; 8 – Mediterranean.

Almost a half of the woodlice species on the Abrau Peninsula are characteristic of Mediterranean ecosystems (Fig. 3). The most widely distributed among Mediterranean species are the four eastern Atlantic species, *Buchnerillolittoralis*, *Trichoniscuspygmaeus*, *Tyloseuropaeus*, and *Halophilosciacouchii*, and also an eastern Mediterranean–Central Asian species, *Chaetophilosciahastata*. Another six species are endemic to Mediterranean ecosystems: *Acaeroplastesmelanurus*, *Armadillidiumvulgare*, *Armadilloofficinalis*, *Platyarthruscaudatus*, *Porcellionidespruinosus*, and *Trachelipusrazzautii*. *Halophilosciacouchii*, *A.vulgare* and *P.pruinosus* originated in the Mediterranean region but are now cosmopolitan.

Another four species in the fauna are widespread: one European (*Buddelundiellacataractae*), one originally European–Asia Minorian but now cosmopolitan (*Cylisticusconvexus*), one originally eastern Palearctic but now Holarctic (*Platyarthrushoffmannseggii*) and one cosmopolitan (*Armadilloniscusellipticus*).

Some of the species are recorded for the first time from the Russian woodlice fauna: *B.cataractae*, *A.melanurus*, *P.caudatus*, A.cf.marmoratum, and *T.europeus*. For two species, the westernmost extent of their ranges are reported from the Abrau Peninsula: *C.giljarovi*, previously known from the northern Caucasus, and *T.lutshniki*, which had been found only in the environs of Sochi.

According to the ecological needs, the 25 species of Abrau Peninsula may be classified into seven groups: hydrophilic, mesophytic, xerophilic, halophilic, myrmecophilic, synanthropic, and euryoecious. *Ligidiumfragile*, *T.pygmaeus*, *C.taitii*, *H.danicus*, *T.pygmaeus*, and *B.littoralis* can be described as hydrophilic inhabitants of freshwater surroundings. *Armadilloniscusellipticus*, A.cf.marmoratum, *H.couchii*, and *T.europeus* are coastal halophilic species. Mesophytic *B.cataractae*, *C.convexus*, *C.giljarovi*, *P.dentifrons*, and all trachelipodids are found solely in wooded biotopes on the peninsula. *Chaetophilosciahastata* and *A.officinalis* are xerophilic, and all three platyarthrid species are myrmecophilic. *Armadillidiumvulgare* and *P.krivolutskyi* are euryoecious. The only synanthropic species is *P.pruinosus*.

Three species were recorded from more than 100 observations each: *Armadillidiumvulgare*, *P.krivolutskyi*, and *T.razzautii*. *C.hastata*, *L.fragile*, *T.utrishensis*, and *T.pygmaeus* were less common but still found in over 40 locations were. *Armadilloofficinalis*, *A.ellipticus*, and *H.danicus* were discovered at 15–20 locations. All myrmecophilic species and 75% of halophilic species were found to be uncommon in our surveys. Other ecological groupings are well represented, ranging from rare to common species.

## ﻿Discussion

The terrestrial isopod fauna of the Abrau Peninsula is quite diverse on a national scale. In our study area, 25 species were found, while the fauna of the former USSR has been reported to have as many as 192 species (Kuznetsova and Gongalsky 2012).

Compared to terrestrial isopod faunas in other Mediterranean and sub-Mediterranean regions, the species diversity on the Abrau Peninsula is not very high. For example, the fauna of Liguria is 97 species (Gardini and Taiti 2023); of the Mediterranean islands, 176 species (Gentile and Argano 2005); and of the Aegean islands, 69 species (Sfenthourakis 1996). Despite the relatively low species diversity, the Abrau Peninsula woodlice fauna is quite rich in endemics of the Caucasus and the peninsula.

The majority of the faunal finds were in June and July. These are the most popular months for soil zoology investigations, when the peninsula is still wet but also warm enough. However, species of isopods can reach their population peak at different times of the year (Warburg 1987). Thus, it seems reasonable that future studies be undertaken in other months. Every few years, one or more species are added to the fauna, indicating that species saturation has not yet been reached.

There is a bias towards natural habitats in the dataset. Many species common in settlements were seldom recorded (e.g. *P.pruinosus*). So, another approach to studying terrestrial isopods of the Abrau Peninsula is to examine buildings and cellars in local settlements.

## ﻿Additional information

### Author ORCIDs

Daria M. Kuznetsova https://orcid.org/0000-0002-1237-1066

Konstantin B. Gongalsky https://orcid.org/0000-0003-1600-8448

### Data availability

All of the data that support the findings of this study are available in the main text.
